# Relationships between support provision, social cohesion and belonging, and well-being among community-dwelling older people: a longitudinal survey study

**DOI:** 10.1186/s12877-026-07908-7

**Published:** 2026-07-02

**Authors:** Wenran Xia, Robbert Huijsman, Jeroen D. H. van Wijngaarden, Martina Buljac- Samardžić

**Affiliations:** Erasmus School of Health Policy and Management, Erasmus University Rotterdam, Rotterdam, 3062 PA The Netherlands

**Keywords:** Active ageing, Mutual support, Social cohesion, Social belonging, Community-dwelling older people

## Abstract

**Background:**

Perceived community environment is important for shaping older people's participation in their communities and for their well-being. Social cohesion and social belonging are two important yet distinct dimensions of perceived community environment that may influence support-behaviour and well-being in different ways. However, previous studies often treated both belonging and support behaviour as components of social cohesion. As a result, the associations between social cohesion and belonging with support provision behaviors and well-being among community-dwelling older people remains underexplored. This study aims at addressing this gap.

**Methods:**

A longitudinal survey study design was employed. Data were collected among community-dwelling older people in the Netherlands across two waves with a six-month interval (*N* = 675). Multivariate linear auto-regressions and cross-lagged panel models (CLPMs) were used to explore the longitudinal associations among support provision, social cohesion, social belonging, and well-being. Sensitivity analyses using change-score regressions were conducted to assess robustness at the intra-individual level.

**Results:**

We found that support provision, social cohesion, and social belonging were all positively associated with follow-up well-being. Cross-lagged analyses revealed distinct longitudinal relationships that, social cohesion at T0 was positively associated with increased support provision at T1, whereas social belonging at T0 was not. Sensitivity analyses confirmed that within-person increases in cohesion were associated with increases in support provision, while changes in belonging were not positively related to support changes.

**Conclusions:**

Our study offers a new understanding of how the community environment may influence older people's support behaviors. Findings suggest that social cohesion and social belonging play distinct roles in relation to support provision among older people. Practices aimed at facilitating mutual support and well-being may need to take specific roles of different dimensions of community environment into account.

**Supplementary Information:**

The online version contains supplementary material available at https://doi.org/10.1186/s12877-026-07908-7.

## Introduction

In recent years, the active ageing agenda has increasingly promoted a positive view of ageing, emphasizing that older people can remain active participants within their communities [4, 5]. In response, many countries are developing community-based initiatives to facilitate mutual support among older people within the community [11, 39, 42]. The underlying assumption is that facilitating mutual support within the community not only alleviates the financial strain on health care systems but also creates opportunities for older people to remain active and live independently, ultimately benefiting their well-being [35]. According to the social-ecological perspective, older people’s behaviors are shaped not only by individual characteristics but also by their interactions with social environments [17]. Understanding how the community environment is associated with older people’s support provision behavior and their psychosocial health is important for facilitating the active ageing process. This study focuses specifically on social cohesion and social belonging as key social dimensions of communities and explores their distinct associations with older people's support provision behavior and well-being.

### Distinguishing social cohesion and social belonging

Social cohesion and social belonging are both considered important social characteristics of the community environment. Both concepts have been found to be associated with positive psychosocial outcomes among older people [8, 12, 27]. However, the distinction between these two concepts remains theoretically underdeveloped and empirically under-researched, as previous studies often treat belonging as a component of social cohesion rather than as a distinct dimension [15, 21]. Distinguishing between these concepts is important for exploring how different aspects of the perceived community environment are associated with older people’s behaviors and well-being.

Social cohesion is usually defined as a cognitive component of social capital, indicating the interactional and relational qualities of community life [25]. According to social capital theory, social cohesion is a community characteristic reflected in the levels of trust, norms of reciprocity, and social bonds within the local social structure, and may improve the efficiency of society by facilitating coordinated action [25]. Social relationships within communities generate social resources through reciprocity, engagement, and social connectedness among community members.

In contrast, social belonging is usually conceptualized as an individual and emotional perspective on community. It is considered a component of sense of community, which has four key components: membership (a sense of belonging), influence (being able to make a difference to a group), integration and fulfillment of need (the fulfillment of needs through membership), and shared emotional connection (commitment of shared experiences) [33]. In this context, social belonging refers to individuals' affective attachment to their community and their sense of fitting into their neighborhood environment, reflecting an individual's social identity connected with the community.

In previous studies, the concepts of social cohesion and social belonging have often been used interchangeably because their underlying theoretical foundations partly overlap. However, social cohesion reflects outward-oriented social interactions and focuses on how individuals are socially embedded within reciprocal community networks, whereas social belonging reflects a more inward and affective sense of attachment to place and community. Therefore, although cohesion and belonging are theoretically related and have frequently used together, distinguishing between these two concepts may be important for understanding older people's behavioral and psychosocial outcomes, as they reflect different aspects of the perceived community environment.

### Social cohesion, social belonging, and support provision

While health-related behaviors and the community environment are both considered important for shaping older people's lives, evidence on how support provision behaviorrelates to the perceived community environment remains limited. One reason for this knowledge gap is that previous research has often incorporated support into the broader conceptualization of social cohesion [7, 25]. However, these studies usually focus on support received from the neighborhood rather than support actively provided to others. This study proposes that, while receiving and providing support are interconnected, they should be examined separately. This distinction is important because support flows in opposite directions between receiving and giving. These two processes may be driven by different motivations and thus lead to different outcomes. Including support provision within the concept of social cohesion may therefore confound social cohesion with its potential causes or outcomes (Jenson [23]). In this study, support provision is treated as a distinct behavioral phenomenon rather than as a definitional component of social cohesion.

Social capital theory suggests that social relationships within communities generate social resources through reciprocity, trust, and norms of mutual engagement [25]. A cohesive community characterized by strong social connections and reciprocal expectations may reflect a higher level of social capital [3], reinforcing positive social norms such as being active and contributing to the community, and thus facilitating older people’s participation in productive activities [30, 32]. Previous studies have found that stronger perceived social cohesion is associated with a higher level of volunteering [24], and that participation in physical activities mediates the association between perceived social cohesion and older people's mental health [26, 28]. Similarly, Davis [10] found positive associations between social cohesion and volunteering. Therefore, perceptions of social cohesion may encourage older people to participate in productive community activities such as providing support within their neighborhood.

In contrast, social belonging may not necessarily operate in the same way. Although feeling attached to the neighborhood may contribute to emotional safety and identification with the community, it does not automatically imply active participation in supportive or reciprocal social interactions. Belonging may therefore relate to support provision differently from cohesion. Empirical research on social belonging and older people's support behaviors remains relatively limited, partly because many studies have treated social belonging as part of social cohesion. Related evidence has shown that feelings of place attachment can predict residents' altruism [44]. A study from Canada found that a sense of belonging was associated with higher levels of social participation, especially among women [31]. Another study examined the relationship between sense of belonging, meaningful participation, and well-being, but found no associations between sense of belonging and the participation intensity [19].

### Social cohesion, social belonging, and well-being

Previous research has consistently shown that both social cohesion and social belonging are associated with better well-being among older adults [8, 26, 28]. On the one hand, communities characterized by trust, reciprocity, and supportive social relationships may provide a sense of security and reassurance that help is available if needed, thereby enhancing psychosocial well-being by fostering emotional security and reducing uncertainty [15]. On the other hand, feeling attached to and integrated within one’s neighborhood may contribute to feelings of comfort, identity, and meaning in later life.

Although both cohesion and belonging may benefit well-being, they may not be equally related to community engagement behaviors such as support provision. Distinguishing between these dimensions may therefore help clarify whether different aspects of the perceived community environment are associated with different outcomes in later life, including both psychosocial well-being and supportive actions.

### Research aim

Despite increasing attention to active ageing and community-based mutual support, research remains limited on whether different social dimensions of the perceived community environment are distinctly associated with support provision and well-being among older people. Guided by the social-ecological framework and informed by social capital theory and sense of community theory, this study explores whether social cohesion and social belonging show distinct associations with support provision behavior and well-being among community-dwelling older people, using data from a two-wave longitudinal survey. The following research question guides this study:

How are social cohesion and social belonging longitudinally associated with support provision and well-being?

## Method

### Data

Data for this study were collected through the Longitudinal Internet Studies for the Social Sciences panel (LISS), which is a probability-based online panel comprising over 6,500 individuals from about 4,700 households, selected from the Dutch population register (https://www.lissdata.nl/). Participants of this panel are invited voluntarily to complete a monthly web-based questionnaire, with monetary compensation. Household and respondent demographics are updated monthly. The English version of questionnaire can be found in the Additional file 1.

Panel members aged 65 and above at the first wave of data collection were invited to fill in the questionnaire in July 2024 for wave 1 and in January of 2025 for wave 2. Data collection in wave 1 generated 734 responses, with 732 participants completing the questionnaire (94.5% response rate). Among those, 677 participants who completed the questionnaire of wave 1 joined the data collection in wave 2, of which 675 (93.8%) completed the questionnaire the second time.

### Measures

#### Well-being

The 15-item version of the Social Production Function Instrument of the Level of Well-being (SPF-IL) was used to measure well-being [16, 34]. This scale was developed from the social production function (SPF) theory, which posits that individuals strive to maintain well-being by fulfilling physical and social needs. It has been validated and proved to be reliable in the Dutch older population [9]. The response category ranged from “Never (1)” to “Always (4)”. The well-being score was assessed as the averaged value of all items, with higher scores indicating better well-being. Cronbach’s alpha values of the SPF-IL in this study were 0.852 at T0 and 0.837 at T1, indicating good reliability.

#### Support provision

As mutual support in the community could be seen as a complex concept that covers various helping behaviors, this study assessed support provision as participants’ participation in a wide range of support tasks including both instrumental support and emotional support [37]. We conceptualize support provision as the overall behavior of helping others, including instrumental and emotional support, as they do not represent different underlying traits, but rather differentiated expressions of a single behavioral tendency of helping and participation in the community.

Participants were firstly presented with the main question “In this section, questions will be asked about your experiences with helping people in your neighborhood, that are not your kin. Think about the last 6 months. How often have you given support or helped someone in other ways, who is NOT a family member?” Activities available that could be selected were: 1) volunteering without payment; 2) support with personal care; 3) support with household chores; 4) support with paperwork; 5) support with technology; 6) financial support; and 7) emotional support. Participants were asked to choose the frequency they have participated in each activity from “Never (0)” to “Almost every day (4)”. Frequencies for each activity were summed up to operationalize the intensity of support, a higher score indicating a higher level of support density.

To evaluate the internal consistency of the measure of support provision, Cronbach’s alpha and confirmatory factor analyses (CFA) were conducted separately for both waves. Cronbach’s alpha coefficients indicated acceptable internal consistency (0.747 at T0 and 0.770 at T1). CFA results further supported a single-factor structure with acceptable model fit and moderate-to-strong factor loadings across support activities (see Additional File 2). These results suggest that the included support activities can reasonably be conceptualized as indicators of a broader support provision contrast.

#### Social cohesion

Social cohesion is measured by an eight-item scale that has been used cross-culturally with good validity and reliability in the Netherlands [9, 12, 14, 45]. Participants were asked about their experience within the neighborhood they live in. Example items are: “If I needed advice about something, I could go to someone in my neighborhood”, “I borrow things and exchange favors with my neighbors”, and “I would be willing to work together with others on something to improve my neighborhood”. All items were measured on a 5-point Likert scale from 1 (Strongly disagree) to 5 (Strongly agree). Items were summed up to create the score of social cohesion, with a higher score indicating a higher level of social cohesion, which is in line with other studies [8]. Cronbach’s alpha values of the cohesion were 0.850 at T0 and 0.842 at T1, and for belonging were 0.841 at T0 and 0.852 at T1, indicating good reliability.

#### Social belonging

Social belonging is measured by a seven-item scale that is usually used together with the social cohesion scale. Similarly, participants were asked about their experience within the neighborhood they live in. Example questions for the belonging sub-scale were: “I am attracted to living in this neighborhood” and “I feel like I belong to this neighborhood”. Similar to scores of social cohesion, items were summed up to create a total score of social belonging. Cronbach’s alpha values of the cohesion were 0.841 at T0 and 0.852 at T1, reflecting good reliability.

#### Covariates

We included background characteristics at T0 that have shown to affect social cohesion and well-being in previous studies [10, 30]. Age and years of residence were treated as continuous variables. Income was measured as the log-transferred gross household income in euros. Self-rated health was measured ranging from poor to excellent and treated as a continuous variable [40]. Gender was dichotomized as male versus female, and ethnic background as Dutch versus non-Dutch. Living arrangement was coded as a dummy variable of single versus co-habitation with partner and/or child(ren). Education level was categorized into low, medium, and high levels.

### Analytic strategy

We conducted a non-response analysis. T-tests and Chi-square tests were used to compare the baseline characteristics of the 675 participants who completed questionnaires in both waves with those of participants who only participated in the first wave. Descriptive statistics and bivariate correlations were then examined to explore correlations among the key variables.

To examine how support provision, social cohesion, and social belonging were associated with well-being, multivariate linear autoregressive regression analyses were conducted. In each model, well-being at T1 was regressed on the baseline level of support provision, cohesion, or belonging, while controlling for baseline well-being and covariates. A multicollinearity test indicated no multicollinearity among the variables (VIF ranges from 1.031 to 3.581).

A cross-lagged panel modelling (CLPM) approach based on structural equation modelling (SEM) was applied to examine longitudinal associations among social cohesion, social belonging, support provision, and well-being across two time points. Under the assumptions of synchronous measurements and stationarity, CLPM evaluates the relationships among variables measured repeatedly at different time points and allows to examine the longitudinal relationships among variables [1, 36]. This study used data from two time points (T0 and T1) with a six-month interval to test time-lagged associations. Given the two-wave design, the analyses should be interpreted as examining longitudinal associations rather than causal or mechanistic relationships. The model included autoregressive paths and cross-lagged associations among the key variables across T0 and T1 while adjusting for baseline covariates. Confidence intervals (CIs) based on 5,000 replications were calculated using a bootstrap resampling.

Following the recommendation of [22], several fit indices were used to assess the model fit, including the root mean square error of approximation (RMSEA), the standardized root mean square residual (SRMR), and the comparative fit index (CFI). A CFI value greater than 0.95 is considered as an excellent model fit, although values greater than 0.90 are considered acceptable [22]. RMSEA and SRMR values of 0.06 or lower are considered to reflect a good fit, while values up to 0.08 are considered acceptable.

Traditional CLPMs may partly reflect stable between-person differences rather than within-person change. To assess the robustness of the longitudinal associations observed in the cross-lagged models, additional sensitivity analyses were conducted using change-score regression models. Specifically, differences scores for all key variables between T0 and T1 were used as indicators of intra-individual variation and included in linear regression models. Each model controlled for baseline levels and demographic covariates, examining whether changes in cohesion, belonging, and support provision were associated with changes in the outcome variables. By using difference scores, this method accounts for fixed individual characteristics that do not change over time and helps to distinguish intra-individual changes from stable between-person differences.

Analyses were performed using the Lavaan package in R (version 4.1.2; R Development Core Team) within the RStudio platform. Maximum likelihood estimation was used to handle any non-normality in the sample. While missing values only exist in covariates, we use the full-information maximum likelihood (FIML) technique to account for missing values, which has been shown to be an efficient method of dealing with missing data compared with traditional imputation [13].

## Results

### Demographic characteristics and correlations

Table 1 presents descriptive characteristics of variables in both waves. Participants in the first wave were aged from 65 to 96 years with a mean age of 73.807 (SD = 5.814) years. Approximately half of the participants were males (52.869%). Almost 70 percent of the participants cohabitated with a partner and/or children (69.130%). Most of the participants were from a Dutch ethnic background (88.243%). Participants have resided in their neighborhood for an average of 28.012 years (SD = 16.922). Characteristics of the sample represent the Dutch population well (Statline [38]). Comparisons of the baseline characteristics of participants who completed questionnaires in both waves to those who only participate in the first wave showed no significant difference, indicating no dropout bias in this study (see Additional File 3).

**Table 1:** Descriptive statistics of participants in both waves

Variable	T0 (*N* = 732)		T1 (*N* = 675)	
	%/range	Mean (SD)	%/range	Mean (SD)
Age	65–96	73.807 (5.814)	65–96	73.799 (5.732)
Gender				
Male	52.869		53.926	
Female	47.131		46.074	
Living arrangement				
Single	30.870		30.538	
Cohabitation with partner and/or child(ren)	69.130		69.462	
Education level				
Low	32.148		32.195	
Medium	30.232		30.119	
High	37.620		37.685	
Self-rated health	1–5	3.014 (0.838)	1–5	3.058 (0.862)
Ethnic groups				
Dutch	88.243		88.243	
Other	11.757		11.757	
Income	0–4.570	3.564 (0.401)	0–4.332	3.566 (0.412)
Years of residence	0–80	28.012 (16.922)	0–80	28.473 (17.355)
Support provision	0–28	3.342 (3.584)	0–28	3.492 (3.847)
Social cohesion	8–40	27.160 (5.577)	9–40	27.250 (5.549)
Belonging	9–35	27.742 (4.101)	11–35	27.964 (4.232)
Well-being	1–4	2.599 (0.423)	1–4	2.648 (0.406)

Correlation analyses show that both age and gender were not significantly associated with either support provision, social cohesion, nor well-being (*p* > 0.05). Income was positively associated with social cohesion (*r* = 0.085*, p* < 0.05 at T0, *r* = 0.140, *p* < 0.001 at T1) but not belonging, support provision, nor well-being. Higher education levels were associated with higher levels of support provision *(r* = 0.114, *p* < 0.01 at T0, *r* = 0.132, *p* < 0.001 at T1) and well-being (*r* = 0.128, *p* < 0.001 at T0, *r* = 0.104, *p* < 0.01 at T1). Co-habiting with partner and/or children and having higher levels of self-rated health were associated with higher levels of cohesion, belonging, and well-being (*p* < 0.001). As indicated in Table 2. all key variables are positively correlated with each other.

**Table 2:** Correlation matrix of key variables

	Support provision		Cohesion		Belonging		Well-being	
	T0	T1	T0	T1	T0	T1	T0	T1
Support provision								
T0		0.587***	0.325***	0.257***	0.148***	0.107**	0.205***	0.169***
T1			0.286***	0.318***	0.147***	0.098*	0.186***	0.244***
Cohesion								
T0				0.803***	0.458***	0.406***	0.481***	0.404***
T0					0.430***	0.484***	0.447***	0.470***
Belonging								
T0						0.758***	0.458***	0.401***
T1							0.404***	0.432***
Well-being								
T0								0.711***
T1								

Correlations among key variables were tested with Pearson correlation statistics. *** *p* < 0.001, ** *p* < 0.01, * *p* < 0.05

### Longitudinal associations with well-being

Table 3 presents results of the auto-regressive analyses examining the longitudinal associations of support provision, social cohesion, social belonging with later well-being. After controlling for baseline well-being and background characteristics, support provision at T0 was positively associated with well-being at T1 with small effect size (*β* = 0.066, p = 0.023, 95% CI: [0.001, 0.014]). Similarly, baseline social cohesion (*β* = 0.118, *p* < 0.001, 95% CI: [0.004, 0.013]) and belonging (*β* = 0.097, *p* = 0.003, 95% CI: [0.003, 0.016]) were both positively associated with later well-being, although the effect sizes are relatively modest. These findings suggest that both the interactional and affective dimensions of perceived community characteristics may be associated older people's well-being over time, providing preliminary evidence for further CLPM explorations.

**Table 3:** Results of linear auto-regressive models

Variable	**Well-being T1**								
	Model 1			Model 2			Model 3		
	B (SE)	*β*	95% CI	B (SE)	*β*	95% CI	B (SE)	*β*	95% CI
Age	0.003* (0.002)	0.042	[−0.001 0.007]	0.003 (0.002)	0.041	[−0.001 0.007]	0.003 (0.002)	0.038	[−0.001 0.007]
Gender (Female)	−0.019 (0.025)	−0.024	[−0.067 0.029]	−0.005 (0.025)	−0.006	[−0.053 0.044]	−0.012(0.025)	−0.014	[−0.060 0.037]
Years of residence	−0.001 (0.001)	−0.006	[−0.002 0.001]	0.000 (0.001)	−0.012	[−0.002 0.001]	0.000 (0.001)	−0.014	[−0.002 0.001]
Income	0.012 (0.029)	0.012	[−0.046 0.069]	0.006 (0.029)	0.006	[−0.052 0.063]	0.015 (0.029)	0.016	[−0.042 0.073]
Health	0.092*** (0.015)	0.188	[0.062 0.121]	0.097*** (0.015)	0.200	[0.068 0.127]	0.089*** (0.015)	0.182	[0.060 0.118]
Ethnic (Dutch)	0.016 (0.037)	0.012	[−0.056 0.089]	0.008 (0.037)	0.006	[−0.064 0.080]	0.020 (0.037)	0.015	[−0.053 0.092]
Education (Low)									
Medium	0.014 (0.031)	0.016	[−0.046 0.074]	0.015 (0.030)	0.017	[−0.045 0.074]	0.021 (0.030)	0.024	[−0.039 0.081]
High	0.021 (0.031)	0.025	[−0.039 0.081]	0.024 (0.030)	0.029	[−0.035 0.083]	0.028 (0.030)	0.033	[−0.032 0.087]
Living Arrangement (single)	0.055 (0.026)	0.062	[0.003 0.106]	0.042 (0.026)	0.047	[−0.010 0.093]	0.042 (0.026)	0.048	[−0.009 0.094]
Wellbeing T0	0.613*** (0.031)	0.625	[0.553 0.673]	0.566*** (0.035)	0.578	[0.498 0.634]	0.585*** (0.033)	0.597	[0.520 0.650]
Support Privision_T0	0.008* (0.003)	0.066	[0.001 0.014]						
Cohesion T0				0.009***(0.002)	0.118	[0.004 0.013]			
Belonging T0							0.010** (0.003)	0.097	[0.003 0.016]
Intercept	0.440 (0.200)		[−0.047 0.832]	0.374 (0.200)		[−0.017 0.765]	0.285 (0.205)		[−0.118 0.688]
R^2^	0.550			0.556			0.554		
Adjusted R^2^	0.541			0.548			0.545		

*B* Unstandardized coefficients,* SE* Standard error, *β* Standardized coefficient.*** p < 0.001, ** p < 0.01, * p < *0.05*

### Longitudinal associations among cohesion, belonging, and support provision

The built model demonstrated good model fit indices (CFI = 0.995, RMSEA = 0.044, SRMR = 0.027). The chi-square test was statistically significant (*χ*^*2*^ = 13.995, *df* = 6, *p* = 0.008), which is common as its value tends to increase along with the sample size [2].

**Fig. 1:**
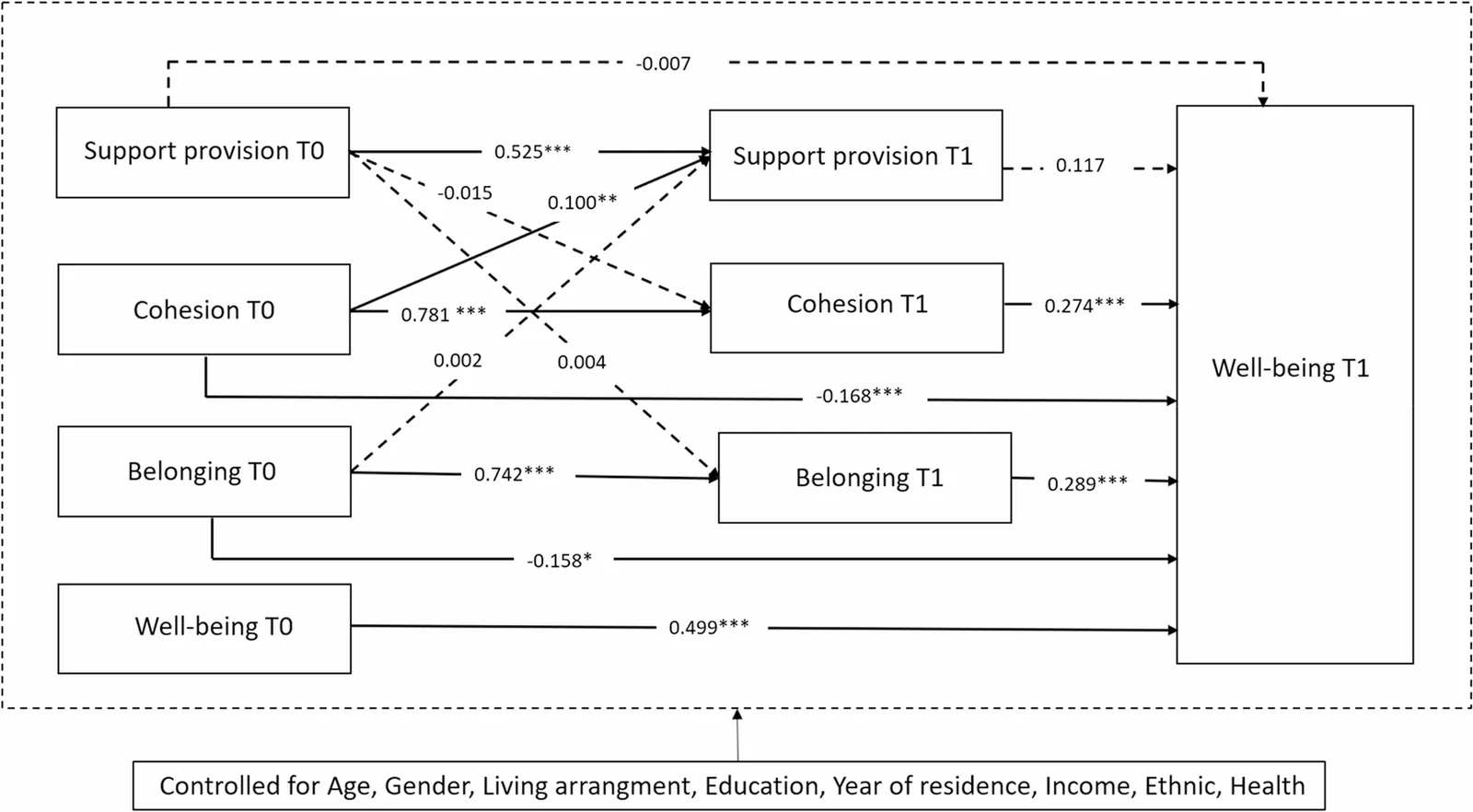
Results of cross-lagged model. (*** *p* < 0.001, ** *p* < 0.01, * *p* < 0.05)

Figure 1 illustrates the estimates for the main paths among key variables. The solid lines represent statistically significant paths among interested variables, and the dotted line represents an insignificant relationship. Numbers on each line showed the standardized coefficients of the path it represents. All auto-regressive paths were statistically significant with large effect sizes (*p* < 0.001), suggesting substantial stability over time for support provision, social cohesion, social belonging, and well-being.

Regarding longitudinal associations between community characteristics and support provision, social cohesion at T0 was positively associated with support provision at T1 (*β* = 0.100, *p* = 0.009, 95% CI [0.017, 0.122]) with small effect size. In contrast, social belonging at T0 was not significantly related with support provision later on (*β* = 0.002, *p* = 0.962, 95% CI [−0.059, 0.068]), indicating an separated relationship of cohesion and belonging with support provision. In addition, support provision at T0 was not significantly associated with either cohesion (*β* = −0.015*, p* = 0.747, 95% CI [−0.162, 0.106]) or belonging (*β* = 0.004, *p* = 0.888, 95% CI [−0.064, 0.072]) at T1. Taken together, these results indicate that cohesion and belonging may show distinct longitudinal relationships with the behavior of support provision. Whereas cohesion was associated with later support provision, belonging did not demonstrate similar associations over time.

### Sensitivity analyses

To examine the robustness of the longitudinal associations between social cohesion and support provision, we conducted additional change-score regression analyses. The results of these analyses were consistent with those of the main model. Within-person increases in social cohesion were significantly and positively associated with within-person increases in support provision from T0 to T1 (*B* = 0.208, *p* < 0.001, *95% CI* [0.128, 0.289]), whereas changes in belonging showed a small negative association that was marginally significant (*B* = −0.117, *p* < 0.05, *95% CI* [−0.217, −0.018]). This finding was consistent with the asymmetric finding observed in the CLPM. Changes of support provision, social cohesion, and social belonging were all positively associated with changes in well-being, suggesting that the difference between cohesion and belonging may be more evident in relation to support behavior. Furthermore, baseline support provision was not significantly associated with changes in cohesion (*B* = 0.067, *p* > 0.05, *95% CI* [−0.017, 0.151]). These within-person change analyses provide additional support for the findings from the CLPMs. Details of the sensitivity analyses can be found in Additional File 4.

## Discussion

By distinguishing social cohesion and social belonging as related but conceptually distinct dimensions of the perceived community environment, this study contributes to the understanding of active ageing and the facilitation of mutual support among community-dwelling older people. Different from previous literature that treats cohesion and belonging as interchangeable indicators of community social characteristics, our findings suggest that these dimensions may relate differently to behavioral and psychosocial outcomes among older people. Specifically, while both cohesion and belonging were longitudinally associated with older people’s well-being, only cohesion was associated with later support provision behavior. These findings suggest that interactional and affective dimensions of the perceived community environment may serve different social functions in later life.

Our results showed that support provision was positively associated with follow-up well-being among older people, although the effect size was relatively modest after controlling for baseline well-being. This finding is consistent with previous research suggesting that providing support outside the family benefits older people’s psychosocial health [6, 43]. Providing support to others may enhance feelings of usefulness and thus increases their self-esteem, which is particularly important in later life [29, 43]. Similarly, both social cohesion and social belonging were positively associated with later well-being. These findings are also in line with previous studies suggesting that perceived supportive community environment contributes to psychosocial well-being among older people [8, 26, 28]. Perceived community-level resources, such as trust and friendliness, may provide older people a sense of reassurance that they can rely on their neighbors when needed [15]. Meanwhile, feeling as a member in the community may serve as an alternative source of social identity, which may further provide a sense of continuity in later life, and thus sustain older people’s well-being [33].

A central finding of this study is the asymmetric longitudinal relationship between the two community dimensions and support provision. Older people who perceived greater social cohesion in their community were more likely to report providing support to others. The finding is consistent with previous research suggesting that perceived social cohesion is associated with older people’s participation in altruistic and productive activities [10, 24]. In contrast, no similar relationship was observed between social belonging and support provision. This finding was observed not only in the cross-lagged model but also in the within-person change-score analyses, in which increases in perceived cohesion were positively associated with increases in support provision, whereas changes in belonging showed no positive association. This asymmetric finding supports our conceptual argument that, social cohesion and social belonging may represent different dimensions of the community social environment. Social cohesion reflects the perceived interactional and relational qualities of the community environment. Older people living in cohesive communities may therefore be more likely to participate in supportive activities because they are socially embedded within reciprocal community networks that encourage engagement and active contribution.

In contrast, social belonging seems to reflect a more inward, affective and identity-oriented attachment to the community environment. It can be influenced by personal experiences and may not always be related to direct social interactions in the community [15]. Interestingly, while the main CLPM analysis showed no significant association between social belonging and support provision, the change-score sensitivity analyses revealed a weak but statistically significant negative within-person association between changes in belonging and changes in support provision. One possible interpretation is that, when older people's needs for affective security and identity are increasingly fulfilled through a sense of belonging, the psychological motivation to provide support to others may correspondingly decrease, as it may reflect a shift of one's status from striving to fit into the community toward feeling embraced by the community. However, given that this finding was observed only in sensitivity analyses with a modest effect size, it should be interpreted very cautiously. Future research is worth conducting to further explore this relationship.

Our findings also contribute to discussions on how community-based mutual support can be facilitated within community environments. Compared with perspectives that emphasize shared identity or homogeneity as the basis of cohesive communities, the findings of this study are more in line with perspectives that discuss social cohesion through reciprocal social interactions and interdependence among community members [18]. From this perspective, supportive community environments may not necessarily emerge because residents share identical identities or strong emotional attachments, but rather because communities foster reciprocal interactions, trust, and opportunities for engagement. Our findings, therefore, suggest that practices aimed at facilitating community-based mutual support may be more closely related to the interactional and relational qualities of community life than to homogeneity or shared identity alone.

### Limitations

Several limitations should be considered when interpreting the findings of this study. First, although this study employed longitudinal survey data, the two-wave design with a six-month interval limits the ability to make strong causal inference or capture long-term dynamic processes. The observed longitudinal associations should therefore not be interpreted as causal relationships. Future studies with longer follow-up periods and additional measurement waves are needed to better capture the temporal dynamics among community characteristics, support provision, and psychosocial well-being among older people. Second, although traditional cross-lagged panel models allow examining temporal ordering, they may partly conflate stable between-person differences with within-person change processes [20]. To address this concern, we conducted additional change-score regressions as sensitivity analyses, which showed consistent findings with the main models. Nevertheless, future studies with more than two waves of data collection that allow the use of random-intercept cross-lagged models may provide more reliable insights into these dynamic processes. Finally, since this study relied on self-reported data, reporting bias and shared method variance may exist. To capture the actual support interactions, other study designs that allow for direct observations, such as action research or mixed methods approaches, are worth conducting to validate the findings based on self-reported data.

### Implications for policy and practice

Findings of this study have implications for policies and practices aiming to facilitate ageing in place and community-based mutual support, although these implications should be interpreted cautiously given the modest effect sizes and the two-wave design. The active ageing agenda advocates for optimizing the ageing process by facilitating older people’s participation within their communities (WHO [41]). Our findings suggest that, facilitating mutual support among older people may require promoting interactional atmosphere in the community. Practically, community-level interventions aimed at strengthening neighborhood interaction and reciprocal relationships may contribute to both supportive engagement and psychosocial well-being among older adults. Examples may include community initiatives that facilitate neighborhood interaction, intergenerational programs, and even the careful use of technologies to promote neighborhood connections. Meanwhile, as both cohesion and belonging were positively associated with well-being, policies promoting emotional attachment within neighborhoods may also remain important for supporting older people’s psychosocial health. Distinguishing these dimensions may therefore help policymakers and practitioners better identify which aspects of community life are most relevant for different ageing-related outcomes.

## Conclusion

To our knowledge, this is the first study to examine whether social cohesion and social belonging show distinct longitudinal associations with support provision and well-being among community-dwelling older people. Using two-wave longitudinal data, our findings indicated that both social cohesion and belonging were positively associated with later well-being. However, only social cohesion was associated with later support provision behavior, whereas social belonging was not. These findings suggest that the interactional and affective dimensions of the perceived community environment may serve different social functions in later life. Distinguishing these dimensions may therefore help advance understanding of how perceived community environments are related to behavioral and psychosocial outcomes among older people. Further longitudinal research using longer follow-up periods is needed to better understand these relationships over time.

## Supplementary Information

Supplementary Material 1.

Supplementary Material 2.

Supplementary Material 3.

Supplementary Material 4.

## Data Availability

The data that support the findings of this study are available from the LISS Data Archive but restrictions apply to the availability of these data, which were used under license for the current study, and so are publicly available. Data are however available from the authors upon reasonable request and with permission of the LISS Data Archive.
