# Supplementary material for: Relationships between support provision, social cohesion and belonging, and well-being among community-dwelling older people: a longitudinal survey study
Source: BMC Geriatr. 2026 Jul 2;26:1205. doi: 10.1186/s12877-026-07908-7 (PMC13613666; doi:10.1186/s12877-026-07908-7)
Supplement: Supplementary file 1 [file 12877_2026_7908_MOESM1_ESM.docx]

**Additional file 1**

**Questionnaire**

**Dear Participants:**

Thank you for participating in this survey. We are conducting research exploring the relationship between informal support and older adults’ health outcomes. We would love to hear from you about your experience regards to this. Your answer is valuable for facilitating a more ageing friendly community.

This questionnaire will take between 5 and 10 minutes to complete. You can stop answering this questionnaire at any time you want. We ensure that your information and answers are completely anonymous and would not be used for any business purposes.

How is your health in general?

□ Excellent

□ Very good

□ Good

□ Fair

□ Poor

How long have you been living in this neighborhood (in years)?

□________

In this section, questions will be asked about your experiences with helping people in your neighborhood, that are not your kin. Think about the last 6 months. How often have you given support or helped someone in other ways, who is NOT a family member?

| **Tasks** | **Never** | **Hardly ever** | **Almost every month** | **Almost every week** | **Almost every day** |
| --- | --- | --- | --- | --- | --- |
| - Volunteering without payment | 0 | 1 | 2 | 3 | 4 |
| - Support with personal care (e.g. dressing, bathing or showering, eating, getting in and out of bed, going to the toilet/bathroom) | 0 | 1 | 2 | 3 | 4 |
| - Support with household chores (e.g. repairs to the house, gardening, transport, doing groceries, household chores) | 0 | 1 | 2 | 3 | 4 |
| - Support with paperwork (e.g. filling out forms, taking care of financial or legal matters) | 0 | 1 | 2 | 3 | 4 |
| - Support with using technology (e.g. phones, computers | 0 | 1 | 2 | 3 | 4 |
| - Financial support (e.g. buying stuff/gifts, giving cash) | 0 | 1 | 2 | 3 | 4 |
| - Emotional support (e.g. providing company, counseling, giving comfort) | 0 | 1 | 2 | 3 | 4 |

**In this section, you will be asked questions of your experience in your neighbourhood. To what extent do you agree or disagree with the following statements?**

| **Question** | **Strongly disagree** | **Disagree** | **Neither agree or disagree** | **Agree** | **Strongly agree** |
| --- | --- | --- | --- | --- | --- |
| 1. I visit my friends at their homes | 1 | 2 | 3 | 4 | 5 |
| 2. The friendships and associations I have with other people in my neighbourhood mean a lot to me | 1 | 2 | 3 | 4 | 5 |
| 3. If I need advice about something I could go to someone in my neighbourhood | 1 | 2 | 3 | 4 | 5 |
| 4. I believe my neighbours would help in an emergency | 1 | 2 | 3 | 4 | 5 |
| 5. I borrow things and exchange favours with my neighbours | 1 | 2 | 3 | 4 | 5 |
| 6. I would be willing to work together with others on something to improve my neighbourhood | 1 | 2 | 3 | 4 | 5 |
| 7. I rarely have a neighbour over to my house to visit | 1 | 2 | 3 | 4 | 5 |
| 8. I regularly stop and talk with people in my neighbourhood | 1 | 2 | 3 | 4 | 5 |
| 9. Overall, I am attracted to living in this neighbourhood | 1 | 2 | 3 | 4 | 5 |
| 10. I feel like I belong to this neighbourhood | 1 | 2 | 3 | 4 | 5 |
| 11. Given the opportunity, I would like to move out of this neighbourhood | 1 | 2 | 3 | 4 | 5 |
| 12. I plan to remain a resident of this neighbourhood for number of years | 1 | 2 | 3 | 4 | 5 |
| 13. I like to think of myself as similar to the people who live in this neighbourhood | 1 | 2 | 3 | 4 | 5 |
| 14. Living in this neighbourhood give me a sense of community | 1 | 2 | 3 | 4 | 5 |
| 15. Overall, I think this is a good place to bring up children | 1 | 2 | 3 | 4 | 5 |

**In this section, we will ask you a number of questions about how you feel. These questions refer to the past 3 months. For your answer, will you please choose between NEVER, SOMETIMES, OFTEN or ALWAYS? If you HARDLY EVER have that feeling you can answer NEVER. If you ALMOST ALWAYS feel that way, answer ALWAYS.**

| **Questions** | **Never** | **Sometimes** | **Often** | **Always** |
| --- | --- | --- | --- | --- |
| 1. Do people pay attention to you? | 1 | 2 | 3 | 4 |
| 2. Do people help you if you have a problem? | 1 | 2 | 3 | 4 |
| 3. Do you feel that people really love you? | 1 | 2 | 3 | 4 |
| 4. There are situations in which we deal with groups of people, for example at home, at work or during our leisure time. Do others appreciated your role in the group? | 1 | 2 | 3 | 4 |
| 5. Do people find you reliable? | 1 | 2 | 3 | 4 |
| 6. Do you feel useful to others? | 1 | 2 | 3 | 4 |
| 7. Do people think you do better than others? | 1 | 2 | 3 | 4 |
| 8. Do people find you an influential person? | 1 | 2 | 3 | 4 |
| 9. Are you known for the things you have accomplished? | 1 | 2 | 3 | 4 |
| 10. In the past few months have you felt relaxed? | 1 | 2 | 3 | 4 |
| 11. In the past few months have you felt perfectly healthy? | 1 | 2 | 3 | 4 |
| 12. In the past few months have you felt physically comfortable? | 1 | 2 | 3 | 4 |
| 13. Are your activities challenging to you? | 1 | 2 | 3 | 4 |
| 14. Do you really enjoy your activities? | 1 | 2 | 3 | 4 |
| 15. How often are you fully concentrated when doing something? | 1 | 2 | 3 | 4 |

Thank you for your participation.
