# Supplementary material for: Relationships between support provision, social cohesion and belonging, and well-being among community-dwelling older people: a longitudinal survey study
Source: BMC Geriatr. 2026 Jul 2;26:1205. doi: 10.1186/s12877-026-07908-7 (PMC13613666; doi:10.1186/s12877-026-07908-7)
Supplement: Supplementary file 2 [file 12877_2026_7908_MOESM2_ESM.docx]

**Additional file 2**

**CFA analysis for variable support provision**

We conducted a confirmatory factor analysis (CFA) to test whether the heterogeneous behavior of support provision pointed to a common construct of “support provision”. Weighted least squares mean and adjusted variance estimator (WLSMV) was used to handle the ordinal categorical data. The single-factor model showed a good to acceptable fit at both time points (see Table A1). All standardized factor loadings ranged from 0.653 to 0.805, and all items were strongly correlated with latent factors (see Table A2). In summary, the seven helping behavior types can be reasonably considered as a single-dimensional indicator of “support provision”, Therefore, in the subsequent analysis, we aggregated the seven items to form the total support provision score.

**Table A1**. Model fit for CFA analysis.

| **Indicator** | **T0** | **T1** | **Reference** |
| --- | --- | --- | --- |
| CFI | 0.982 | 0.982 | > 0.95 (good) |
| TLI | 0.972 | 0.973 | > 0.95 (good) |
| RMSEA | 0.067 | 0.077 | < 0.08 (acceptable) |
| SRMR | 0.075 | 0.071 | < 0.08 (good) |

**Table A2**. Standardized factor loading for support provision items.

| **Item** | **Factor loading_T0** | **Factor loading_T1** |
| --- | --- | --- |
| Volunteering | 0.690 | 0.688 |
| Support with personal care | 0.726 | 0.743 |
| Support with household chores | 0.762 | 0.750 |
| Support with paperwork | 0.737 | 0.805 |
| Support with using technology | 0.680 | 0.731 |
| Financial support | 0.653 | 0.676 |
| Emotional support | 0.695 | 0.714 |
