# Supplementary material for: Relationships between support provision, social cohesion and belonging, and well-being among community-dwelling older people: a longitudinal survey study
Source: BMC Geriatr. 2026 Jul 2;26:1205. doi: 10.1186/s12877-026-07908-7 (PMC13613666; doi:10.1186/s12877-026-07908-7)
Supplement: Supplementary file 3 [file 12877_2026_7908_MOESM3_ESM.docx]

**Additional file 3**

Comparison of background characteristics of participants who completed questionnaires of both waves to those who only participate in the first wave.

| Variable | T0 (N=732) | | T1 (N=675) | | P value  (t-test/  Chi-square) |
| --- | --- | --- | --- | --- | --- |
|  | %/range | Mean (SD) | %/Range | Mean (SD) |  |
| Age | 65-96 | 73.81 (5.81) | 65-96 | 73.80 (5.73) | 0.90 |
| Gender |  |  |  |  | 0.06 |
| Male | 52.87 |  | 53.93 |  |  |
| Female | 47.13 |  | 46.07 |  |  |
| Living arrangement |  |  |  |  | 0.13 |
| Single | 29.10 |  | 28.30 |  |  |
| Cohabitation with partner and/or child(ren) | 70.90 |  | 71.70 |  |  |
| Education level |  |  |  |  | 0.71 |
| Low | 32.15 |  | 32.19 |  |  |
| Medium | 30.23 |  | 30.12 |  |  |
| High | 37.62 |  | 37.69 |  |  |
| Self-rated health | 1-5 | 3.01(0.84) | 1-5 | 3.02(0.83) | 0.58 |
| Ethnic groups |  |  |  |  | 1.00 |
| Dutch | 88.24 |  | 88.24 |  |  |
| Other | 11.76 |  | 11.76 |  |  |
| Income | 0-4.57 | 3.564 (0.40) | 0-4.332 | 3.57 (0.41) | 0.27 |
| Years of residence | 0-80 | 28.01 (16.92) | 0-80 | 28.47 (17.36) | 0.71 |
| Support provision | 0-28 | 3.34 (3.58) | 0-28 | 3.49 (3.85) | 0.39 |
| Social cohesion | 8-40 | 27.16 (5.58) | 9-40 | 27.25 (5.55) | 0.59 |
| Belonging | 9-35 | 27.74 (4.10) | 11-35 | 27.96 (4.23) | 0.37 |
| Well-being | 1-4 | 2.60 (0.42) | 1-4 | 2.65 (0.41) | 0.66 |
