# Supplementary material for: Relationships between support provision, social cohesion and belonging, and well-being among community-dwelling older people: a longitudinal survey study
Source: BMC Geriatr. 2026 Jul 2;26:1205. doi: 10.1186/s12877-026-07908-7 (PMC13613666; doi:10.1186/s12877-026-07908-7)
Supplement: Supplementary file 4 [file 12877_2026_7908_MOESM4_ESM.docx]

**Additional file 4 Sensitivity Analyses**

**Change-score regression analyses**

The table provides full results of the change-score regressions reported as sensitivity analyses in the main manuscript. Each model regress within individual change scores from T0 to T1 for key variables. Three separate linear regression models were estimated, with change of support provision, change of cohesion, and change of well-being as dependent variables, controlling for covariates. The results confirm the main finding of this study: at the within-person level, changes in social cohesion were positively associated with change in support provision (B = 0.208, p< 0.001). In addition, baseline cohesion is also positively associated with support provision change (B = 0.113, p<0.001). In contrast, neither baseline belonging nor changes in belonging is positively associated with support provision change. Both cohesion and belonging changes are positively associated with changes in well-being, and the baseline support provision is not significantly associated with cohesion change. These results confirm findings in cross-lagged panel model, support the robustness of the main conclusions.

**Table 1.** Results of change-score regressions

| Variable | Change_Support provision | | Change_Cohesion | | Change_Well-being | |
| --- | --- | --- | --- | --- | --- | --- |
|  | B (SE) | 95% CI | B (SE) | 95% CI | B (SE) | 95% CI |
| Support provision_T0 | -0.440*** | [-0.517, -0.364] | 0.067 | [-0.017, 0.151] | 0.013*** | [0.005, 0.020] |
| Cohesion_T0 | 0.113*** | [0.051, 0.174] | -0.265*** | [-0.323, -0.207] | 0.008** | [0.003, 0.013] |
| Belonging_T0 | -0.034 | [-0.112, 0.044] | 0.138*** | [0.061, 0.215] | 0.010** | [0.003, 0.016] |
| Well-being_T0 | 0.087 | [-0.706, 0.881] | 0.456 | [-0.335, 1.246] | -0.475*** | [-0.542, -0.407] |
|  |  |  |  |  |  |  |
| Change_Support provision | - | - | 0.207*** | [0.127, 0.288] | 0.015*** | [0.008, 0.022] |
| Change_Cohesion | **0.208***** | [0.128, 0.289] | - | - | 0.015*** | [0.008, 0.022] |

**Continued Table 1.** Results of change-score regressions

| Variable | Change_Support provision | | Change_Cohesion | | Change_Well-being | |
| --- | --- | --- | --- | --- | --- | --- |
|  | B (SE) | 95% CI | B (SE) | 95% CI | B (SE) | 95% CI |

| Change_Belonging | -0.117* | [-0.217, -0.018] | 0.298*** | [0.202, 0.395] | 0.015*** | [0.006, 0.023] |
| --- | --- | --- | --- | --- | --- | --- |
|  |  |  |  |  |  |  |
| Age | -0.017 | [-0.063, 0.028] | -0.005 | [-0.051, 0.040] | 0.003 | [-0.001, 0.006] |
| Gender (Female) | 0.100 | [-0.445, 0.645] | 0.190 | [-0.354, 0.733] | -0.005 | [-0.052, 0.041] |
| Years of residence | 0.007 | [-0.008, 0.023] | -0.003 | [-0.019, 0.012] | 0.000 | [-0.002, 0.001] |
| Income | 0.101 | [-0.543, 0.744] | 0.634 | [-0.006, 1.273] | -0.014 | [-0.069, 0.040] |
| Health | 0.045 | [-0.285, 0.376] | 0.266 | [-0.063, 0.595] | 0.089*** | [0.061, 0.117] |
| Ethnic (Dutch) | 0.123 | [-0.684, 0.929] | -0.153 | [-0.957, 0.652] | 0.018 | [-0.051, 0.086] |
| Education (Low) |  |  |  |  |  |  |
| Medium | 0.185 | [-0.481, 0.851] | -0.085 | [-0.749, 0.580] | 0.018 | [-0.039, 0.074] |
| High | 0.115 | [-0.550, 0.781] | 0.153 | [-0.511, 0.817] | 0.016 | [-0.041, 0.072] |
| Living Arrangement (single) | -0.555 | [-1.135, 0.025] | -0.063 | [-0.644, 0.518] | 0.051* | [0.002, 0.100] |
| Intercept | 0.023 | [-4.467, 4.514] | -0.630 | [-5.111, 3.850] | 0.290 | [-0.091, 0.672] |
| R^2^ | 0.226 |  | 0.226 |  | 0.318 |  |
| Adjusted R^2^ | 0.205 |  | 0.205 |  | 0.299 |  |

*Notes. B = unstandardized regression coefficient; CI = confidence interval. *** p<0.001, ** p<0.01, * p<0.05.*
